# The rare case of synchronous bilateral breast metastasis from a lung neuroendocrine tumor (small cell lung carcinoma): a case report and literature review

**DOI:** 10.1186/s40792-024-01877-y

**Published:** 2024-09-18

**Authors:** Ayaka Shimo, Koichiro Tsugawa, Kaori Sakamaki, Mina Kitajima, Mariko Takishita, Mizuho Tazo, Mari Nakano, Takako Kuroda, Ai Motoyoshi, Makiko Tsuzuki, Toru Nishikawa, Hisanori Kawamoto, Masatomo Doi

**Affiliations:** 1 Department of Breast and Endocrine Surgery, Kawasaki Municipal Tama Hospital, 1-30-37, Syukugawara, Tama, Kawasaki, Kanagawa 214-0021 Japan; 2 Department of Breast and Endocrine Surgery, St. Marianna University School of Medicine, 2-16-1, Sugao, Miyamae, Kawasaki, Kanagawa 216-8511 Japan; 3 Department of Breast and Endocrine Surgery, Breast and Imaging Center, St. Marianna University School of Medicine, 6-7-2, Manpukuji, Asao, Kawasaki, Kanagawa 215-0004 Japan; 4 Department of Diagnostic Pathology, Kawasaki Municipal Tama Hospital, 1-30-37, Syukugawara, Tama, Kawasaki, Kanagawa 214-0021 Japan

**Keywords:** Bilateral, Breast metastasis, Small cell carcinoma, Lung neuroendocrine carcinoma

## Abstract

**Background:**

Breast metastasis from small cell neuroendocrine carcinoma (SNEC) is very rare. In the present report, we describe a case of a female patient who was initially diagnosed with triple negative primary bilateral breast cancer, but during systemic examination, the diagnosis was bilateral breast metastasis from SNEC.

**Case presentation:**

A 62-year-old woman with no history of smoking presented to the Department of General Medicine with left-sided chest pain, and computed tomography revealed masses in both breasts and left pleural thickening that was further confirmed by mammography and ultrasound of the breasts. A needle biopsy was performed, and triple negative primary bilateral breast cancer was diagnosed. Because progastrin-releasing peptide (ProGRP) 37,300 pg/ml (normal range, 0–81.0 pg/ml) and neuron-specific enolase 35.0 ng/ml (normal range, 0–16.3 ng/ml) levels were elevated, thoracoscopic biopsy was performed, and SNEC was diagnosed. Pathological examinations showed that the bilateral breast masses were also positive for immunohistochemical staining of chromogranin A, synaptophysin, and CD56, leading to a diagnosis of bilateral breast metastasis of neuroendocrine tumor.

**Conclusion:**

Although very rare, the possibility of breast metastasis should be considered when malignancy is suspected in other organs.

## Background

Breast cancer is the most commonly diagnosed cancer worldwide, surpassing lung cancer in 2020. The global cancer statistics 2020 estimate that 2.3 million new breast cancer cases were diagnosed, representing 11.7% of all cancer cases. Globally, breast cancer kills approximately 680,000 people annually [1].

Breast cancer easily metastasizes to the bones, liver, lungs, and brain. Conversely, metastasis to the breast is very rare, accounting for 0.4–1.3% of all malignant breast tumors [2–4].

Most reported metastases to the breast are from hematologic malignancies, such as leukemia and lymphoma, with some reports from malignant melanoma, lung, ovarian, prostate, renal, stomach, ileum, and thyroid cancers [4, 5].

In particular, metastasis from small cell neuroendocrine carcinoma (SNEC) is very rare, with few reports in the literature.

In the present report, we describe a case of a female patient who was initially diagnosed with triple negative primary bilateral breast cancer, but during systemic examination, the diagnosis was bilateral breast metastasis from SNEC.

## Case presentation

A 62-year-old woman with no smoking history experienced itching on the left side of her chest 16 months before diagnosis. She visited a dermatologist, but no abnormality was found, and she was followed up with. Seven months later, she fell and bruised her chest. Since then, she had left-sided chest pain when coughing and sneezing. She was prescribed painkillers, but the left-sided chest pain gradually became more severe. She thought it was caused by yoga, which she had recently started practicing. She visited a general practitioner who noted multiple masses in both breasts.

Her only medical history was of scoliosis and chronic gastritis, neither of which required treatment.

She had no family history of cancer, no allergies, no alcohol consumption, or no smoking.

A screening mammogram 6 months earlier revealed no abnormalities.

**Fig. 1:**
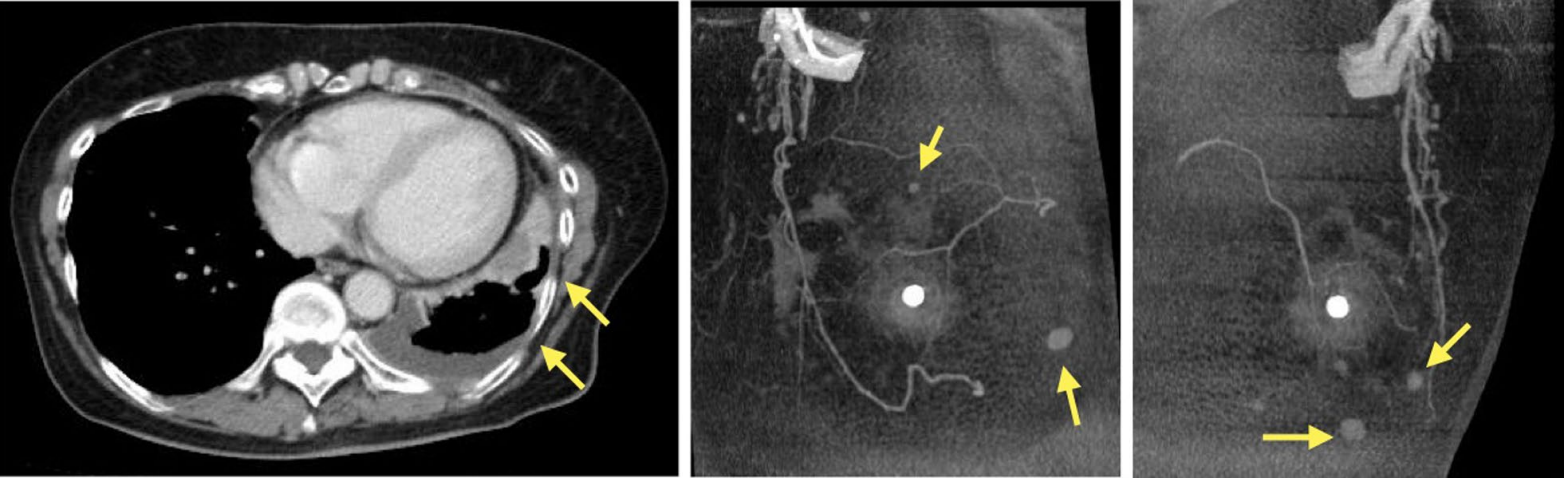
A contrast-enhanced computed tomography (CT) scan revealed irregular pleural thickening and a more tangential mass-like structure at the base of the left lung. Multiple nodules were also revealed in both breasts

A contrast-enhanced computed tomography (CT) scan revealed slightly irregular pleural thickening from the base to dorsal pleura of the left lung and a more tangential mass-like structure at the base of the left lung. There was no abnomality in the hilum. There were no obvious rib fractures. Since multiple nodules were found in both breasts, a consultation with a breast surgeon was recommended (Fig. 1).

On palpation, an elastic, firm, well-defined, slightly poorly mobile mass of approximately 1–2 cm was palpated on the B resion of the right breast and the D resion of the left breast. No axillary or supraclavicular lymph nodes were observed.

**Fig. 2:**
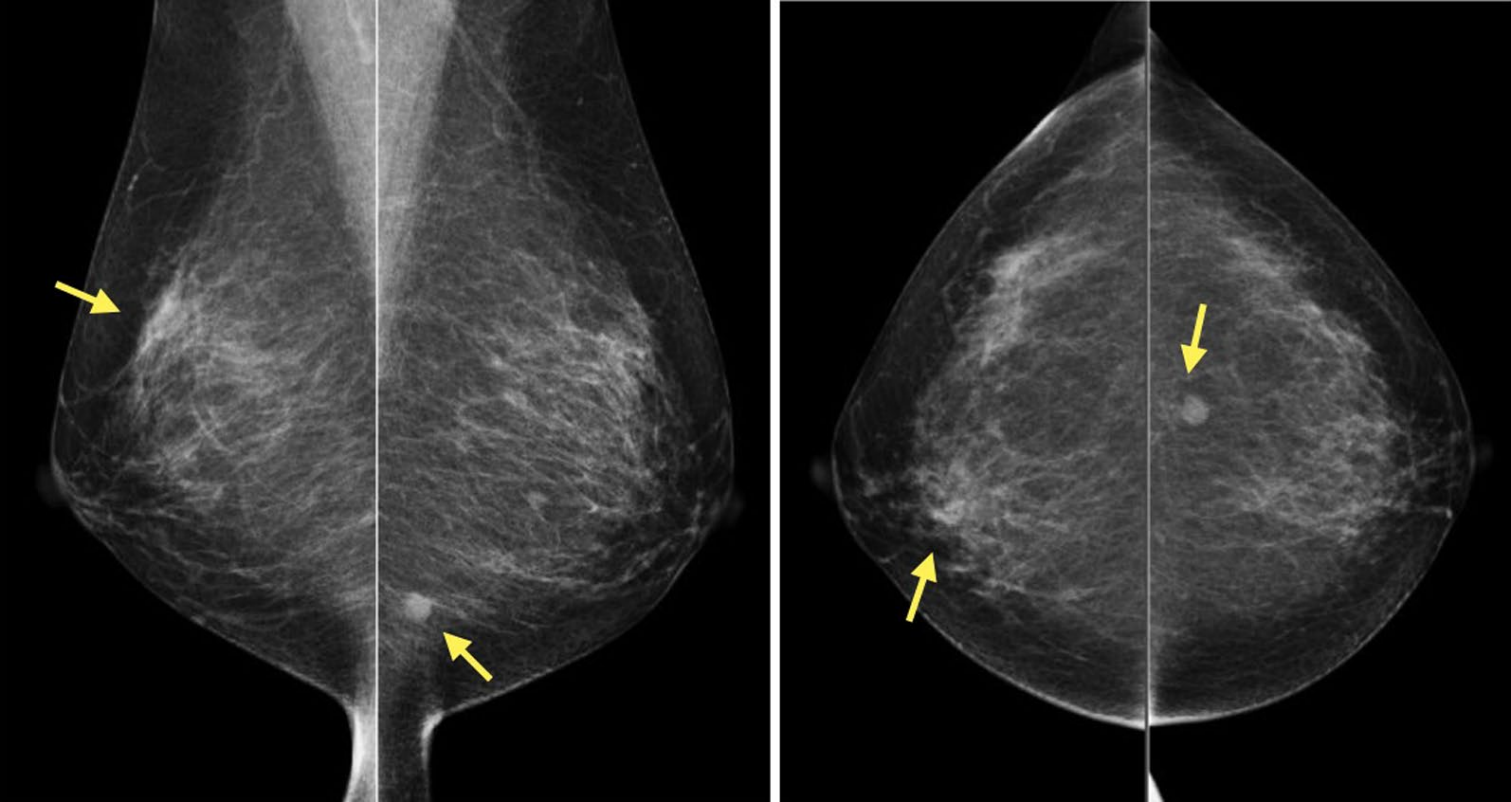
Mammography revealed a round, well-defined, hyperintense mass in the left breast. There was focal asymmetric density in the right breast

Mammography revealed a round, well-defined, hyperintense mass in the left breast without calcification. The mass was not clearly visible in the right breast, but there was focal asymmetric density (Fig. 2).

**Fig. 3:**
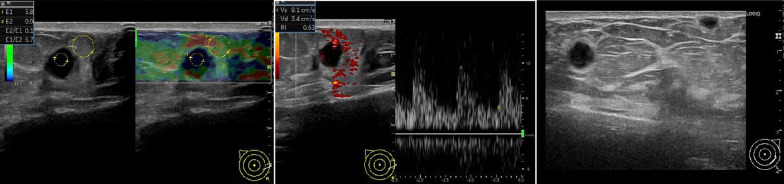
Ultrasonography revealed multiple round, well-defined, full, hypoechoic masses with internal pulsatile blood flow signal in both breasts. Elastography showed relatively uniform firmness

Ultrasonography showed multiple round, well-defined, full, hypoechoic masses with internal pulsatile blood flow signal. Elastography showed relatively uniform firmness. The multiple masses were showed in both breasts’ subcutaneous and mammary tissues. The largest masses were 7 mm in the B restion of the right breast and 6 mm in the D resion of the left breast, and the multiple masses were similar in shape. There were no bilateral axillary lymph node enlargements (Fig. 3).

**Fig. 4:**
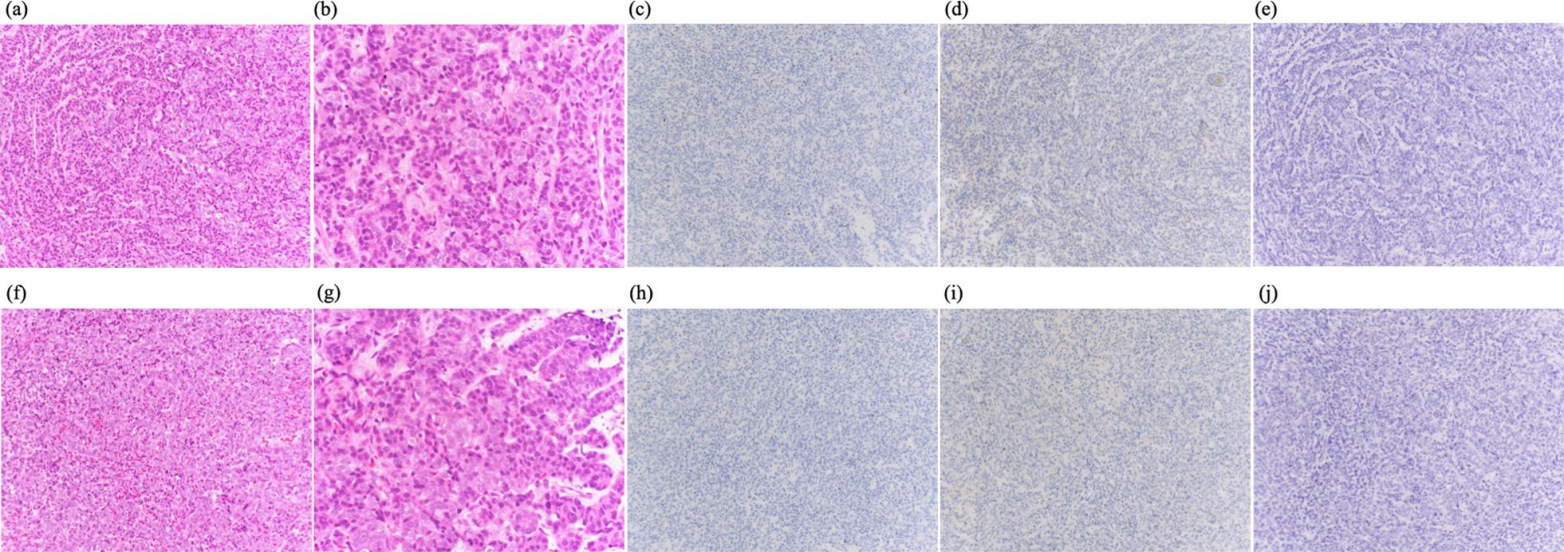
Photomicrographs of the biopsied breast tumor sections. **a**–**e** are the right breast tissue, **f–j** are the left breast tissue. **a**, **f** Hematoxylin and eosin (H &E) -staining (magnification × 20), **b**, **g** (magnification × 40), **c**, **h** Immunohistochemistry of ER (magnification × 20), **d**, **i** PgR (magnification × 20), **e**, **j** HER2 (magnification × 20)

An ultrasound-guided needle biopsy was performed on the B resion of the right breast and the D resion of the left breast. Both breast masses were diagnosed as solid-type invasive ductal carcinoma of the breast. Furthermore, both had the same immunostaining characteristics of triple negative type, with negative estrogen receptor (ER), progesterone receptor (PgR), and human epidermal growth factor receptor2 (HER2) status. The pathology confirmed the diagnosis of triple negative primary bilateral multiple breast cancer (Fig. 4).

**Fig. 5:**
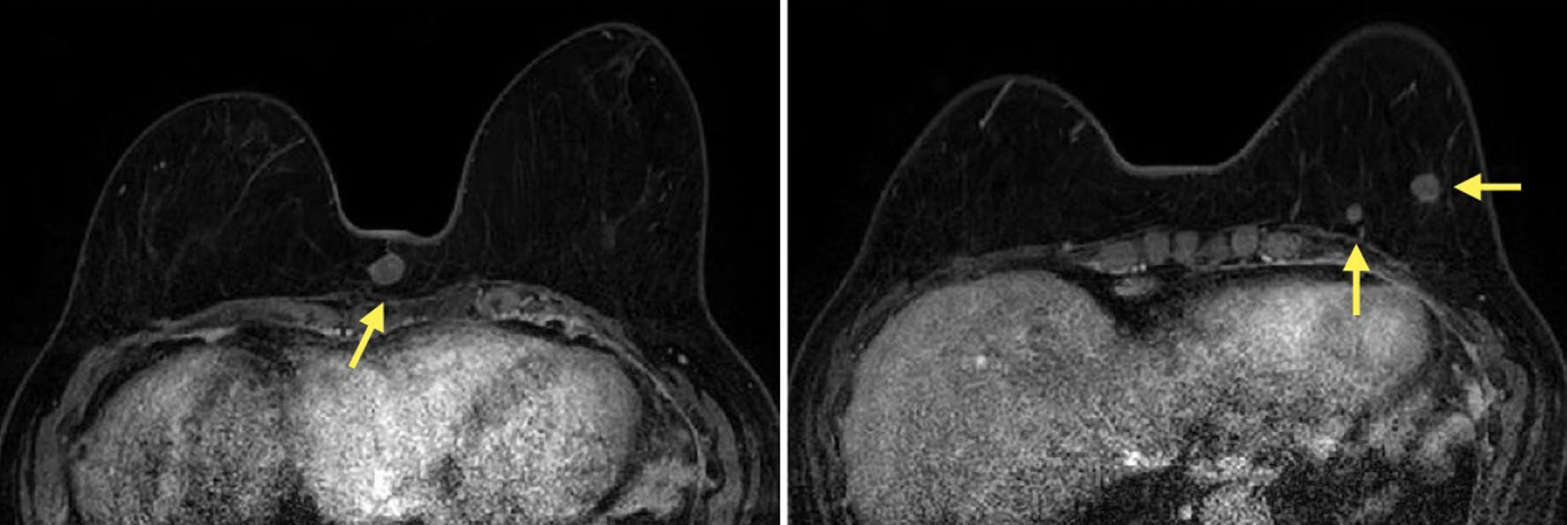
Contrast-enhanced MRI showed multiple masses with rim enhancement and hypo-diffusion in both breasts’ subcutaneous and mammary tissues

Contrast-enhanced MRI showed multiple masses with rim enhancement and hypo-diffusion in both breasts’ subcutaneous and mammary tissues. The largest masses were 13 mm in the B region of the right breast and 10 mm in the D region of the left breast, and all masses, including these masses, showed similar contrast patterns (Fig. 5).

**Fig. 6:**
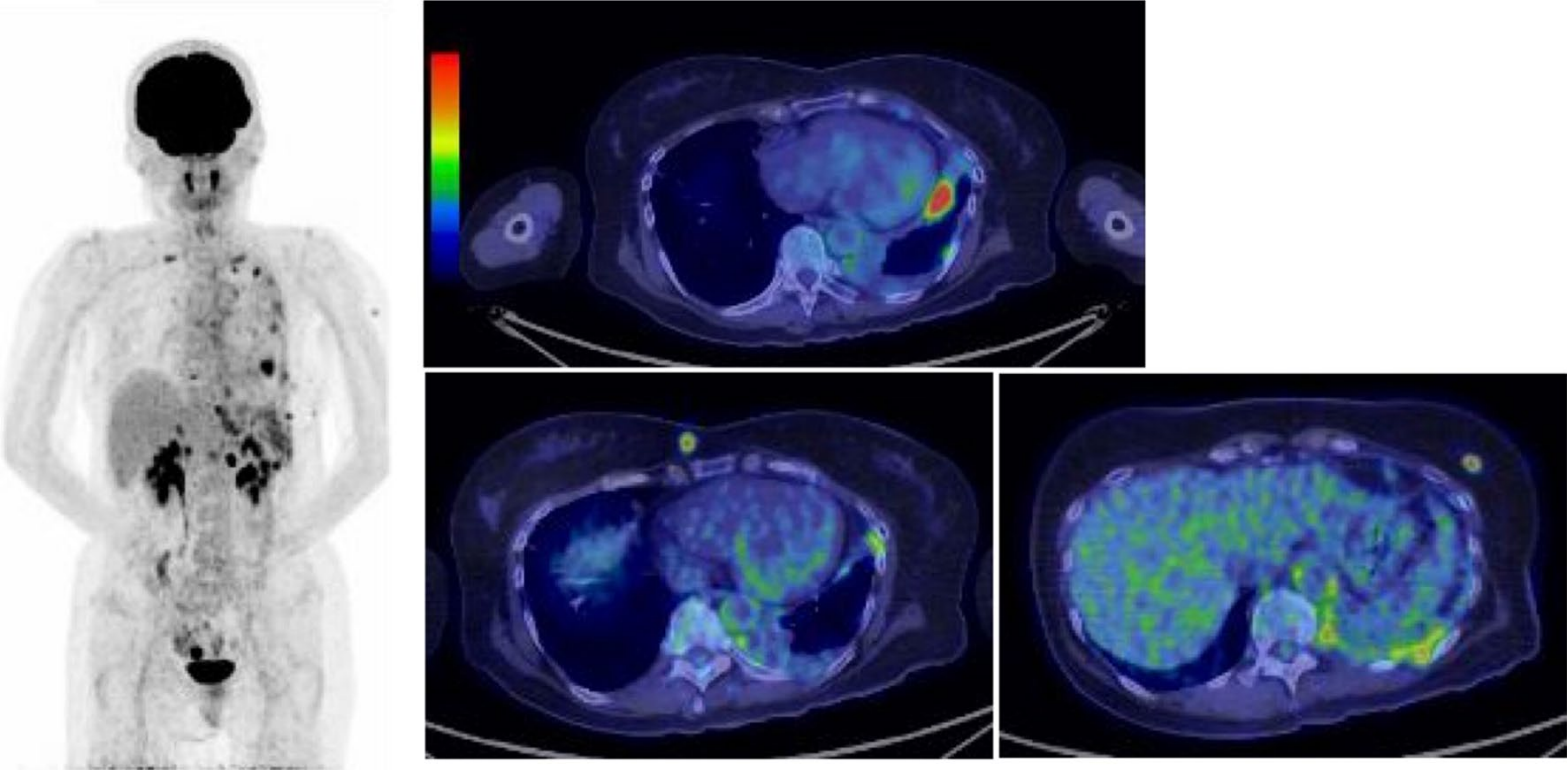
18F-fluorodeoxyglucose (FDG) positron emission tomography-CT showed a nodule with FDG accumulation (SUVmax = 11.42) in the left lower lobe. Multiple FDG accumulations were also observed in the bilateral breasts

18F-fluorodeoxyglucose (FDG) positron emission tomography-CT showed a nodule with FDG accumulation (SUVmax = 11.42) in the left lower lobe. In addition, multiple other nodules were also found in the bilateral breasts, such as a 1.1 cm nodule (SUVmax = 6.27) in the left D region and a 1.1 cm nodule (SUVmax = 5.75) in the right B region in the breast subcutaneous tissue. Multiple FDG accumulations were also observed in the left pleura, diaphragm, supraclavicular fossa lymph node, and left parasternal lymph node. Notably, no significant FDG accumulations were observed in subcutaneous tissue other than the breasts (Fig. 6).

Blood tumor markers were high: progastrin-releasing peptide (ProGRP) 37,300 pg/ml (normal range, 0–81.0 pg/ml) and neuron-specific enolase (NSE) 35.0 ng/ml (normal range, 0–16.3 ng/ml). Carcinoembryonic antigen (CEA), carbohydrate antigen 15–3 (CA15-3), squamous cell carcinoma (SCC), sialyl Lewis X-i antigen (SLX), and cytokeratin fragment (CYFRA) were within the normal ranges.

**Fig. 7:**
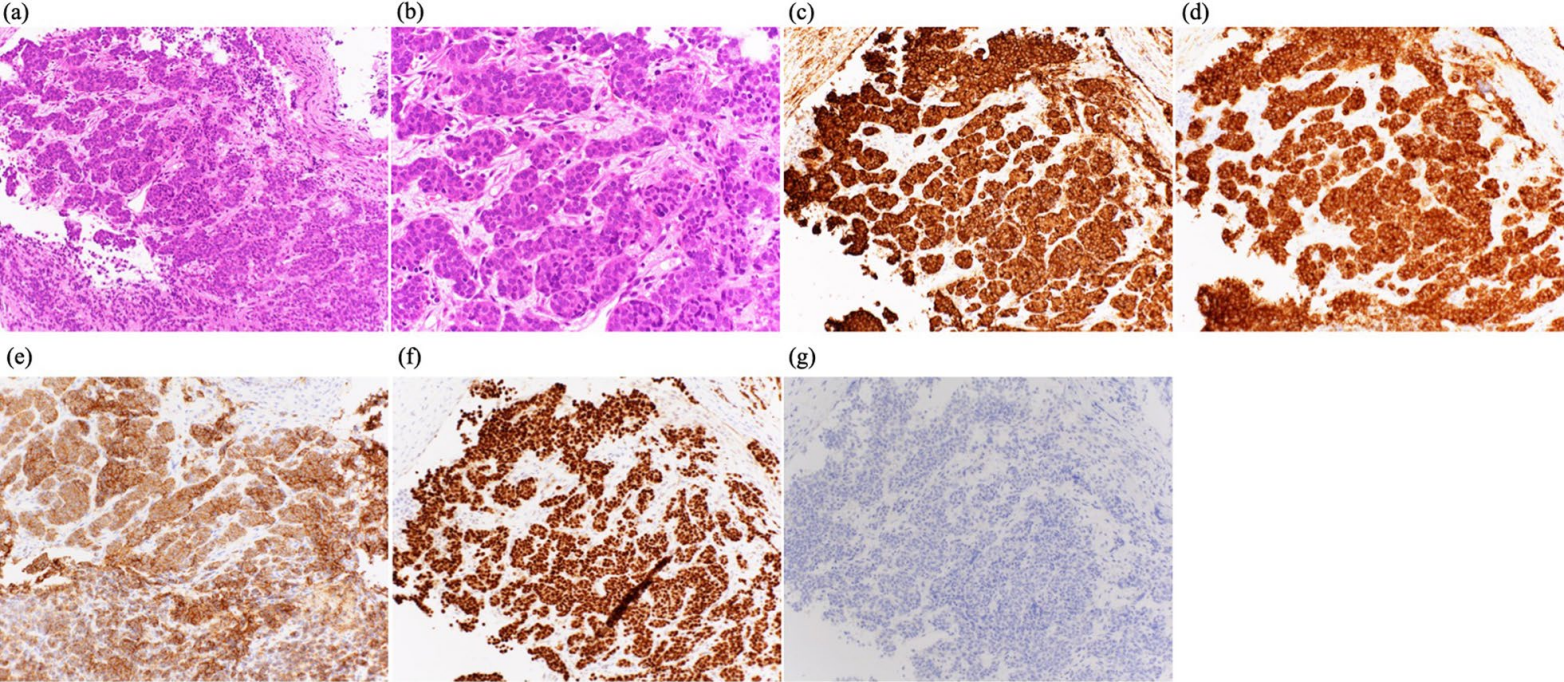
Photomicrographs of the biopsied lung lesion. **a** Hematoxylin and eosin (H &E) -staining (magnification × 20), **b** (magnification × 40), **c** Immunohistochemistry of ChromograninA (magnification × 20), **d** Synaptophysin (magnification × 20), **e** CD56 (magnification × 20), **f** thyroid transcription factor-1-TTF-1 (magnification × 20), **g** p40 (magnification × 20)

Based on high ProGRP levels, we considered the possibility of primary lung cancer and performed a thoracoscopic biopsy. Pathological examination revealed atypical epithelial cells in a full or sporulated fashion, small cells with a high N/C ratio, and slightly larger cells with eosinophilic cytoplasm. ChromograninA ( +), synaptophysin ( +), CD56 ( +), thyroid transcription factor-1-TTF-1 ( +), and p40 (−) were the immunostaining characteristics of the cancer. Based on the above findings, the diagnosis of SNEC was made (Fig. 7).

**Fig. 8:**
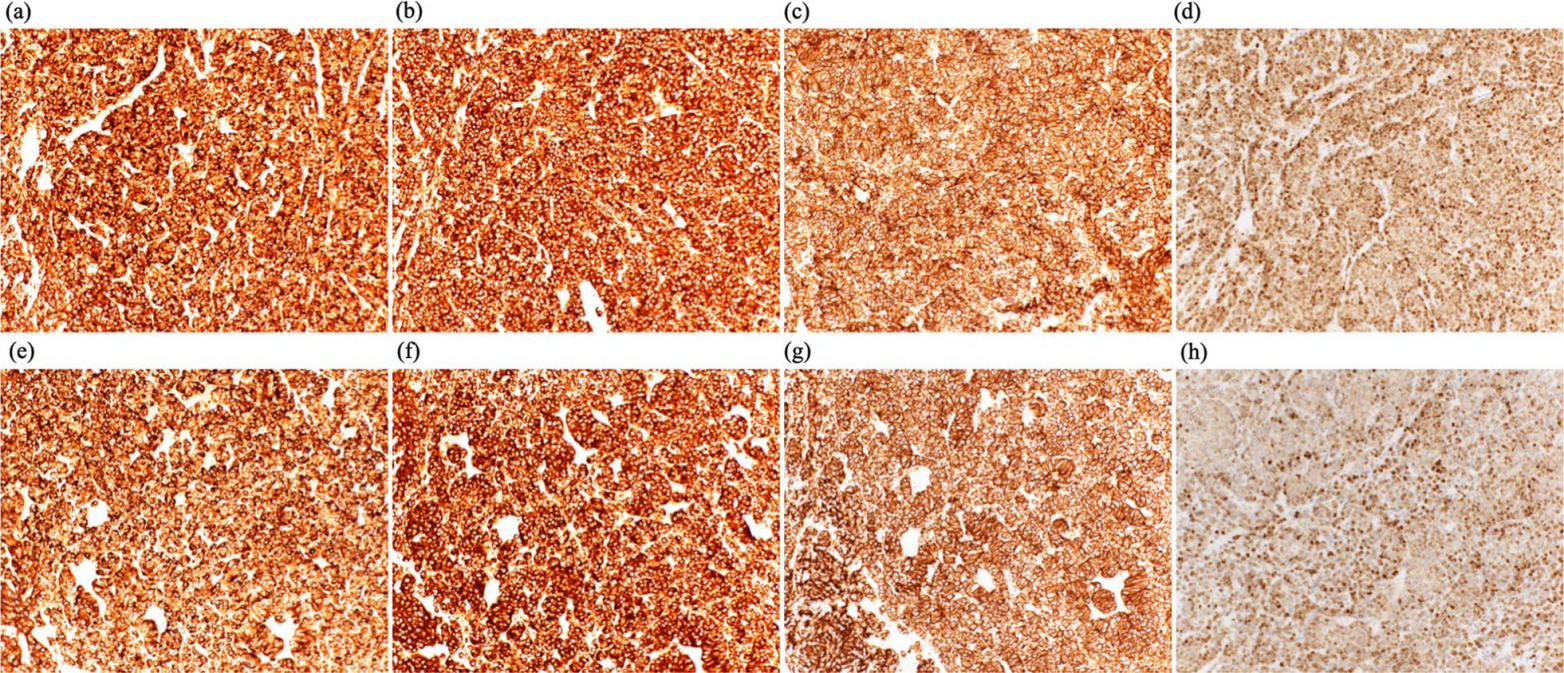
Photomicrographs of the biopsied breast tumor sections. **a**–**d** are the right breast tissue, **e**–**h** are the left breast tissue. **a**, **e** Immunohistochemistry of ChromograninA (magnification × 20), **b**, **f** Synaptophysin (magnification × 20), **c**, **g** CD56 (magnification × 20), **d**, **h** thyroid transcription factor-1-TTF-1 (magnification × 20)

Additional immunostaining was performed on needle biopsies of bilateral breast masses (chromograninA ( +), synaptophysin ( +), CD56( +), thyroid transcription factor-1-TTF-1 ( +), cytokeratin AE1/AE3 ( +), LCA(−), E-cadherin ( +), napsin A (−), ER = 0%, PgR = 0%, HER2 = 0, Ki-67 = 30%). The immunostaining results of the bilateral breast masses were the same. Furthermore, the lung tissue from thoracoscopic biopsy also showed the same findings (Fig. 8).

The patient was finally diagnosed with bilateral breast metastases of primary SNEC. The patient was started on cisplatin, etoposide, and atezolizumab in the Department of Respiratory Medicine to treat stage IV small cell lung cancer.

## Discussion

Small cell lung cancer is common in smokers but rare in nonsmokers. Small cell lung cancer is a high-grade neuroendocrine tumor known to be more aggressive than other lung cancers. Approximately 70% of cases have distant metastases, mainly to the liver, brain, bones, and adrenal glands [3]. Thyroid, ovary, pancreas, stomach, intestine, and pituitary gland metastases have also been reported, although rarely [6].

Breast metastasis of SNEC is very rare. There are very few reports of it in the literature. Furthermore, reports of bilateral breast metastasis are especially rare [3].

One school of thought is that breast metastasis from small cell lung cancer occurs to the ipsilateral breast via lymphatic vessels [7]. The other is that the metastasis is hematogenous since it may also metastasize to the contralateral breast or both breasts [5]. According to Jose et al., only 16% of patients had bilateral metastasis, while the rest had metastasis on only one side [8]. In our case, multiple metastasis were showed in the subcutaneous and mammary tissues in both breasts. No significant masses were observed in subcutaneous tissue other than the breasts. Similar findings have also been reported [9, 10]. We consider that the cancer cells can metastasize to breasts close to the primary tumor through the hematogenous route or a retrograde axillary lymphatic drainage.

A review paper by Joakim Crona et al. reported that breast metastases of neuroendocrine tumors could be identified in 85% of cases by mammography and 100% of cases by ultrasound [7]. The imaging findings of breast metastases are characterized by a round, well-defined, hyperintense mass on mammography without calcification, spicula, or architecture disorganization. On ultrasound, many hypoechoic masses are full or cystic and may be diagnosed as benign. Therefore, they may not proceed to a full pathological examination and are instead followed up with, delaying the diagnosis [3, 4].

Pathological findings of breast metastases are often negative for ER, PgR, and HER2 status, so cases of triple negative breast cancer are often diagnosed differently [5, 7, 9–14].

Immunohistochemical markers such as chromogranin A, synaptophysin, and CD56 are neuroendocrine tumors’ most common positive markers. TTF1 is also an important marker positive in 80% of lung cancers, although it may also be positive in thyroid tumors [4, 15]. In particular, in small cell lung cancer, TTF1 is more likely to be expressed in the peripheral type than in the central type, and is attracting attention as a factor for poor prognosis [16]. However, it is rarely stained routinely in the diagnosis of breast cancer. In our case, chromogranin A, synaptophysin, CD56, and TTF1 were not stained initially, and the diagnosis was triple negative primary bilateral breast cancer. Since ProGRP 37300 pg/ml (normal range, 0–81.0 pg/ml) was abnormally high, a lung biopsy was performed, and a diagnosis of small cell lung cancer was made. The possibility of double carcinoma of the breast and lung remained, but because of the multiple bilateral breast masses, additional staining for neuroendocrine tumor markers was performed, and a diagnosis of breast metastasis was made. A similar diagnosis might not have been made if the breast mass had been unilateral and solitary.

Vaughan et al. reported three cases of breast metastasis from neuroendocrine lung cancer, and surgery was performed in two of them [17]. Similar to the present case, Kotake et al. also reported bilateral breast metastases from neuroendocrine lung cancer in a non-smoker who underwent mastectomy and radiation for local control [2]. The breast mass was not resected in the present case, but local control was good with chemotherapy.

It has been proven that primary resection of breast cancer with distant metastases does not prolong prognosis [18]. However, the benefit of surgical resection of breast metastases has not been established. Some reports suggest that removal of most metastases may be associated with a better prognosis than otherwise [19]. Small cell lung cancer has a very poor prognosis, with a 2-year survival rate of only 8.5% [4]. Therefore, it is essential to diagnose it and initiate treatment at an early stage. In case there is bleeding, pain, or other problems that may reduce quality of life, surgical resection should be limited to local control purposes.

Recently, reports of breast metastasis of neuroendocrine cancer have been increasing. We believe this is due to the increasing awareness of specialists and advances in imaging. Some have suggested that cases of mammary metastasis may have been misdiagnosed as primary breast cancer in the past [13]. Carreras et al. reported a retrospective analysis of metastatic sites of neuroendocrine tumors in 4210 cases using 68 Ga-PET/CT. Their results showed breast metastases in 21 cases (0.5%), which may be the fifth most frequent after liver, lymph node, bone, and heart metastases. Furthermore, they suggested that breast metastasis of neuroendocrine tumors is not rare [20]. Jose et al. summarized 116 cases of breast metastasis from neuroendocrine tumors. The most common primary sites were gastrointestinal (63%), lung (27%), ovarian (3%), renal (1%), cervical (1%), endometrial (1%), and thymic (1%) [8].

In particular, when a triple negative malignancy is found in another organ, the possibility of breast metastasis, although rare, should be taken into consideration, and further examination should be conducted.

## Conclusions

We observed a very rare case of bilateral breast metastasis from SNEC. Although the diagnosis was difficult to make, we were able to provide appropriate treatment after a series of thorough examinations.

## Data Availability

Not applicable.

## Abbreviations

SNEC: Small cell neuroendocrine carcinoma
ProGRP: Progastrin-releasing peptide
CT: Contrast-enhanced computed tomography
ER: Estrogen receptor
PgR: Progesterone receptor
HER2: Human epidermal growth factor receptor 2
FDG: Fluorodeoxyglucose
NSE: Neuron-specific enolase
CEA: Carcinoembryonic antigen
CA15-3: Carbohydrate antigen 15-3
SCC: Squamous cell carcinoma
SLX: Sialyl Lewis X-i antigen
CYFRA: Cytokeratin fragment
